# B cell epitopes on infliximab identified by oligopeptide microarray with unprocessed patient sera

**DOI:** 10.1186/s12967-015-0706-7

**Published:** 2015-10-29

**Authors:** Arne Homann, Niels Röckendorf, Arno Kromminga, Andreas Frey, Uta Jappe

**Affiliations:** Division of Clinical and Molecular Allergology, Research Center Borstel (RCB), Priority Area Asthma and Allergy, Airway Research Center North (ARCN), German Center for Lung Research (DZL), Borstel, Germany; Division of Mucosal Immunology and Diagnostics, Research Center Borstel (RCB), Priority Area Asthma and Allergy, Airway Research Center North (ARCN), German Center for Lung Research (DZL), Borstel, Germany; IPM Biotech, Hamburg, Germany; Interdisciplinary Allergy Division, Department of Internal Medicine, University of Luebeck, Luebeck, Germany

**Keywords:** Adalimumab, Anti-drug-antibody, Biologicals, Epitope mapping, Infliximab, Oligopeptide microarray, Therapeutic monoclonal antibody, TNF-alpha

## Abstract

**Background:**

Autoimmune diseases like rheumatoid arthritis and inflammatory bowel disease are treated with TNF-alpha-blocking antibodies such as infliximab and adalimumab. A common side effect of therapeutic antibodies is the induction of anti-drug antibodies, which may reduce therapeutic efficacy.

**Methods:**

In order to reveal immunogenic epitopes on infliximab which are responsible for the adverse effects, sera from patients treated with infliximab were screened by ELISA for anti-infliximab antibodies. Sera containing high levels of anti-drug-antibodies (>1.25 µg/ml) were analyzed in an oligopeptide microarray system containing immobilized 15-meric oligopeptides from the infliximab amino acid sequence. Immunogenic infliximab IgG-epitopes were identified by infrared fluorescence scanning and comparison of infliximab-treated patients versus untreated controls.

**Results:**

Six relevant epitopes on infliximab were recognized by the majority of all patient sera: 4 in the variable and 2 in the constant region. Three of the epitopes in the variable region are located in the TNF-alpha binding region of infliximab. The fourth epitope of the variable part of infliximab is located close to the TNF-alpha binding region and contains an N-glycosylation sequon. The sera positive for anti-infliximab antibodies do not contain antibodies against adalimumab as determined by ELISA. Thus, there is no infliximab–adalimumab cross-reactivity as determined by these systems.

**Conclusions:**

Our data shall contribute to a knowledge-based recommendation for a potentially necessary therapy switch from infliximab to another type of TNF-alpha-blocker. The characterization of immunogenic epitopes on therapeutic monoclonal antibodies using unprocessed patient sera shall lead to direct translational aspects for the development of less immunogenic therapeutic antibodies. Patients benefit from less adverse events and longer lasting drug effects.

## Background

Therapeutic monoclonal antibodies are a group of biological drugs against diseases which are difficult to treat by “classical” small molecule drugs. These diseases include autoimmune disorders such as inflammatory bowel disease (IBD) and rheumatoid arthritis (RA) [1, 2]. The therapeutic principle of these monoclonal antibodies in both disorders is the blockade of endogenous tumour necrosis factor α (TNF-α). By inhibiting TNF-α, the autoimmune-inflammation cascade is interrupted, and the symptoms of the disease are suppressed [1, 3]. Yet, early on in monoclonal antibody therapy, adverse reactions such as hypersensitivity or reduced efficacy of biological drugs were observed [4–8]. These adverse reactions, induced by anti-drug antibodies (ADA) of different isotypes, were subject of numerous investigations, but the pathomechanisms including the immunological prerequisites that lead to these phenomena are not fully understood. Immediate-type reactions with severe allergic symptoms and even fatal anaphylaxis are reported for cetuximab. In this case, the IgE-mediated pathomechanism involves an immune reaction towards the glycan galactose-α-(1,3)-galactose, termed α-Gal on this chimeric antibody [9]. ADA are also involved in the reduced efficacy of therapeutic antibodies which may result in clinical non-responsiveness [10–12]. These ADA frequently develop during the course of therapy. Furthermore, it has also been shown that there are pre-existing antibodies against biologicals in some individuals [8, 13, 14]. Foreign murine epitopes in the variable region of chimeric antibodies like infliximab (IFX) may well be responsible for the formation of ADA. Moreover, despite the elimination of chimeric epitopes of therapeutic antibodies by so-called antibody humanization, there is still a number of patients who develop ADA [15]. Medical consequences of ADA formation may either be increase of dosage and/or application frequency of the therapeutic antibody, discontinuation of the treatment or a switch to another type of biological drug [16].

In this study, a microarray-based chip system was established that allows the screening of serum IgG from unprocessed patient sera. Immunogenic epitopes in the variable region of infliximab were identified by epitope mapping of 20 sera from infliximab-treated patients. Although there are first reports indicating that neutralizing ADA are directed against the TNF-α binding region of infliximab (IFX) [17], the exact immunogenic sequences in the complementary-determining region of IFX are determined in this study. To address the question of a possible therapy switch, cross-reactivity of the infliximab-reactive sera with an alternative therapeutic antibody against TNF-α (adalimumab, ADL) was investigated.

## Methods

### Patients and untreated controls

Sera of patients included in this study were sent from a clinical laboratory (16 samples) or a gastroenterologist (4 samples). The mean age of the patients was 37 years with 60 % females (Table 1). The mean anti-IFX antibody level was 51 µg/ml. The control group consists of healthy individuals from Northern Germany. 75 % are females (10/15), and the average age of this group is 35. Serum levels of anti-IFX antibodies in the control group were below the detection limit (<1.25 µg/ml).

**Table 1:** Characteristics of IFX-treated patients and untreated healthy controls

	Patients	Untreated controls
Mean age	37 y	35 y
Gender distribution	60 % female	75 % female
Mean ADA level	51 µg/ml	<1.25 µg/ml

*y* years, *ADA* anti-drug antibody

### ELISA measurement of anti-infliximab/anti-adalimumab antibodies in patient sera

The anti-IFX serum antibody titer was determined by a heterogeneous bridging ELISA test (IPM Biotech). For this test, a 96-well microtiter plate (Nunc maxisorp, VWR) was coated with 3 µg/ml of IFX per well for 2 h at 37 °C. After washing 3× with PBS buffer (50 mM, pH 7.4), wells were refilled with 50 µl Tris buffer (1 M, pH 9.5). 100 µl of diluted patient sera (1:10) were acid-treated to dissolve potential immune complexes (100 mM acetic acid, 5 min, RT) and pipetted into the Tris-buffer-containing wells. The microtiter plate was incubated at 4 °C overnight. The following day, the wells were washed 3× with PBS (50 mM) and 65 µl of acetic acid (100 mM) were pipetted into each well. After a 5 min incubation at RT on a shaker, 50 µl of the acid-treated fractions were transferred onto an untreated plate containing 50 µl Tris buffer (1 M, pH 9.5) per well. After an incubation time of 60 min (RT), the wells were washed 3× with PBS-Tween (2.5 % v/v, PBST) and filled with 200 µl blocking buffer (5 % BSA, 5 % Casein in PBST). After another 60 min of incubation at RT and 3× washing with PBST, 10 µl biotinylated IFX (0.25 µg/ml) were added to each well. After 60 min rocking at RT and 3× washing with PBST, 100 µl streptavidin (0.5 µg/ml) conjugated with horseradish peroxidase (HRP) were added to each well, followed by another 15 min rocking at RT and washing 3× with PBST before 100 µl of TMB substrate were added. For colour development, the plate was incubated for 5 min at RT in the dark and the reaction was stopped by addition of 100 µl sulphuric acid (1 M) per well. The plates were analysed by an ELISA reader (Dynex, MRX) at wavelengths of 450 and 630 nm. All analyses were performed in duplicate. Anti-IFX standards (quantification range 80–1.25 µg/ml) were run on each microtiter plate. The analysis of anti-ADL antibodies was performed accordingly except for the use of a biotinylated anti-ADL reagent.

### Analysis of serum rheumatoid factors

IgA, IgM and IgG rheumatoid factors were determined by a commercial ELISA assay according to the manufacturer’s instructions (4027 RF IgA, 4046 RF IgM, 4085 RF IgG, GA Generic Assays).

### Generation of infliximab oligopeptide microarrays

**Fig. 1:**
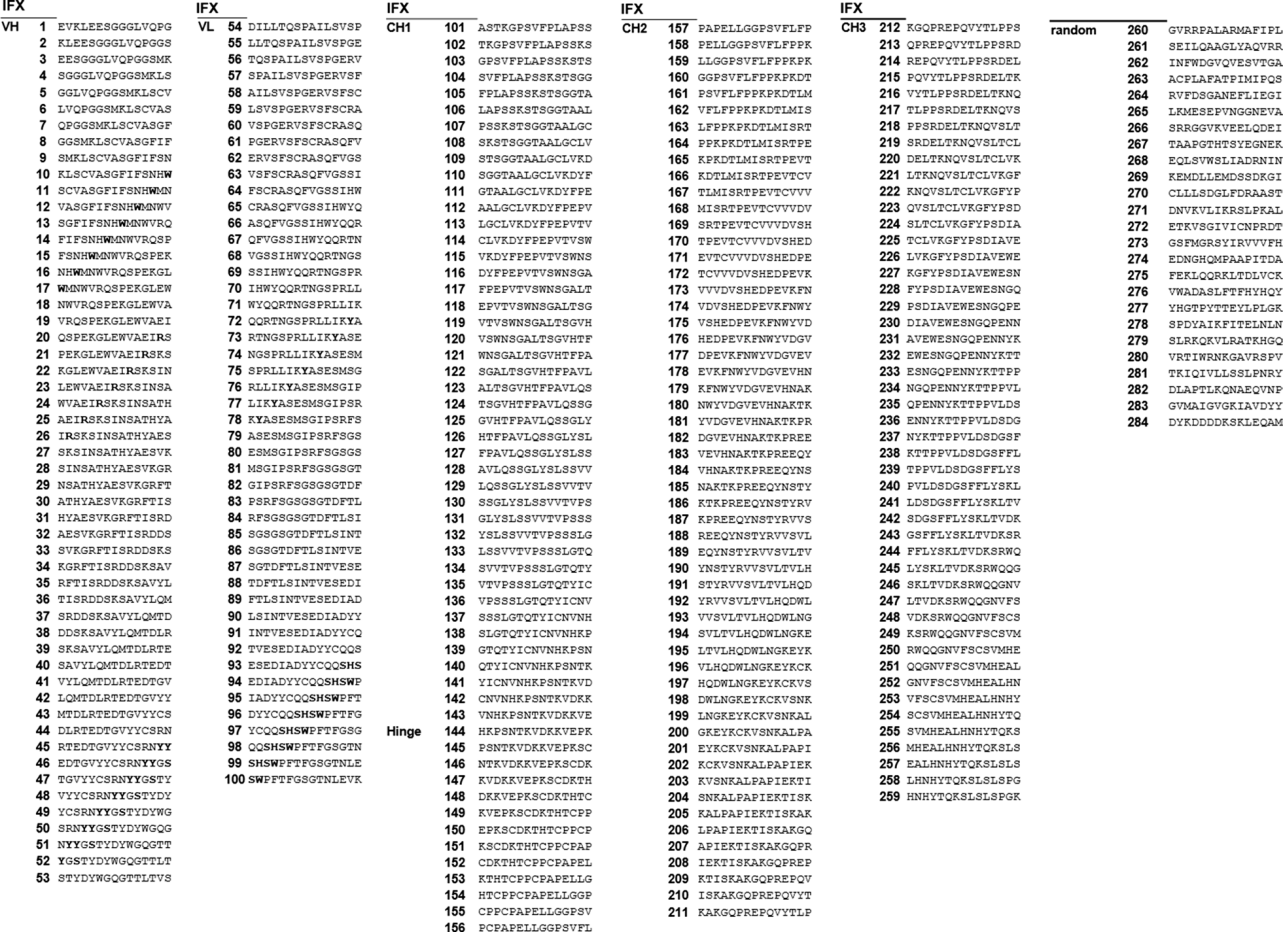
IFX-derived peptides used for oligopeptide array construction and analysed by epitope mapping of IFX-treated patient sera. 15-meric oligopeptides derived from the amino acid sequence of IFX with an offset of two amino-acids and random internal standardization peptides were spotted on cellulose-coated slides

15-mer peptides derived from the amino acid sequence of IFX (drug bank acc. no. DB00065, Fig. 1) were synthesized by Fmoc solid phase synthesis on amine-derivatized cellulose disks of 2.7 mm diameter (Intavis Bioanalytical Instruments AG) using an automated multiple peptide synthesizer (MultiPep RS, Intavis Bioanalytical Instruments AG). For this, the cellulose membrane disks were mounted into 384-well-footprint synthesis frames, and Fmoc protecting groups present on the amine functions of the disks were removed by treating each disk (3×) with 4 µl of 20 % (v/v) piperidine in dimethylformamide (DMF). Subsequent washing of the deprotected disks was performed 4× with 35 µl of DMF and 6× with 50 µl of ethanol each. Fmoc-protected amino acid derivatives were pre-activated by converting them into their corresponding oximes. This was achieved by adding a 1.1 M solution of diisopropyl carbodiimide (DIC) in DMF to a solution containing 0.4 M *N*-α-Fmoc-protected amino acid and 0.7 M ethyl-cyanoglyoxylate-2-oxime (Oxyma Pure, Merck) in DMF. The resulting final concentration was 0.25 M DIC, 0.2 M *N*-α-Fmoc-protected amino acid and 0.35 M Oxyma pure. Coupling of pre-activated amino acid derivatives to cellulose disks was accomplished by applying 1 µl of these solutions to the respective cellulose disks. Coupling at each disk was repeated 3× and a minimum of 30 min of reaction time was allowed in each synthesis cycle. After coupling, unreacted amino groups were capped with 4 µl of 5 % (v/v) acetic anhydride in DMF (5 min, RT) before the next synthesis cycle was started by removal of the Fmoc groups. After completion of the peptide synthesis, the disks were treated 4× for 5 min with 4 µl of a solution of 20 % (v/v) piperidine in DMF, and the liberated amino termini were acetylated 3× for 5 min with 4 µl of a 5 % (v/v) mixture of acetic anhydride in DMF at RT. The disks were washed 7× with 35 µl of DMF followed by six washes with 50 µl of EtOH each before they were air-dried by application of vacuum suction for 12 min and transferred to 96-well plates (MegaBlock 96 well 2.2 ml; Sarstedt). Side chain protecting groups of the peptides were cleaved by treatment with 150 µl of cleavage cocktail (80 % of trifluoroacetic acid, TFA), 12 % of dichloromethane (DCM) 5 % of water and 2.5 % of triisobutylsilane [TIBS) (v/v)] per well for 2 h at RT. Subsequently, the cleavage cocktail was removed, and the disks were treated with 250 µl of cellulose lyse-solution containing 88.5 % of TFA, 4 % of trifluoromethane sulfonic acid (TFMSA) 5 % of water and 2.5 % of TIBS for 10 min on an ultrasound bath and for additional 16 h under continuous shaking. After disintegration of the disks, 750 µl of cold *tert*-butylmethylether (TBME) were added and mixtures were kept at −20 °C for 90 min to precipitate the dissolved matter. Liquids were carefully removed and precipitates were resuspended 2× in 750 µl of MTBE per well. MTBE was removed and the precipitates were dissolved in 500 µl of DMSO per well by treating the suspensions in the microwell plates for 10 min on an ultrasound bath and by an additional 16 h continuous shaking. Plates were centrifuged (2800×*g*, 5 min, Haeraeus Megafuge 1.0 R) for 10 min and 40 µl of the supernatant from each well were transferred to a 384 well microtiter plate. 40 µl of SSC buffer (SSC buffer: 3 M NaCl (175 g/L), 0.3 M sodium citrate·2H_2_O (88 g/L), adjusted to pH 7.0 with 1 M HCl, diluted 1:20 with water) were added to each well, the plates were sealed with an adhesive lid and treated on an ultrasound bath for 5 min. Peptide-modified cellulose solutions were transferred to cellulose-coated glass slides (Intavis Bioanalytical Instruments AG) using a slide-spotting robot (AutoSpot ASP222, Abimed Analysentechnik). A volume of 0.06 µl for each peptide was spotted in duplicate onto the slides in two arrays with 384 positions each (16 × 24 spots, 1.2 × 1.2 mm grid). Slides were air-dried and stored dry at −20 °C.

### Epitope mapping of anti-infliximab serum IgG

To identify IgG binding epitopes on IFX, the oligopeptide microarrays were probed with IFX-reactive patient sera (Table 2). The slides were allowed to thaw for 10 min, rehydrated with 100 % ethanol for 10 min on a horizontal shaker at RT and washed 3× with TBST (Tris-buffered saline, 50 mM, pH 7.4, Tween, 2.5 %) for 10 min. Slides were blocked with casein buffer (5 % in H_2_O) for 4 h under shaking at RT. After washing for 10 min with TBST, patient sera were applied in a dilution of 1:500 in blocking buffer according to previous optimizations of patient serum and secondary antibody concentrations. Serum incubation was performed overnight at 4 °C on a horizontal shaker. Next, the slides were washed 6× for 10 min with TBST. The secondary antibody (anti-human-IgG (H + L), IRDye 800 CW fluorophore, Li-Cor Biosciences GmbH) was applied in a dilution of 1:500 in blocking buffer and incubated for 2 h. After washing 6× for 10 min, the slides were dried and read-out using an infrared imager (Li-Cor, Odyssey, intensity 1, 42 µm resolution, high quality setting). The whole epitope mapping process was performed in three independent experiments in duplicate for the 20 anti-IFX-positive patient sera and the 14 untreated control sera.

**Table 2:** ADA levels in sera from patients treated with IFX and untreated healthy controls

Patient ID	Age	Gender	ADA (µg/ml)	Control ID	Age	Gender	ADA (µg/ml)
438	29	M	54.1	P*	48	F	<1.25
452	30	F	63.9	F10	20	M	<1.25
485	64	F	74.1	F22	25	F	<1.25
522	42	F	45.9	F28	22	M	<1.25
587	41	F	78.4	F31	45	M	<1.25
609	30	M	>80	F32	59	F	<1.25
623	57	F	53.9	F33	25	F	<1.25
626	57	F	44.1	F38	53	F	<1.25
638	32	M	39.3	F41	19	M	<1.25
639	33	M	47.4	F42	32	M	<1.25
648	4	F	34.2	F43	31	F	<1.25
652	31	M	29.7	F44	44	F	<1.25
660	26	M	14.4	F45	36	F	<1.25
681	–	–	24.1	F46	35	F	<1.25
694	13	F	45.6	F47	33	F	<1.25
695	37	M	59.9				
719	16	F	69.7				
721	–	–	>80				
722	42	M	44.8				
724	80	F	32.6				

Specific anti-drug-antibody (ADA) levels gainst IFX were determined by ELISA. The ADA detection range is 1.25–80 µg/ml

–, patient information not available

### Microarray data analysis and statistics

Quantitation of the fluorescence signals was performed using Odyssey, version 2.1.12 (Licor Biosciences). Further data analysis and statistics was conducted with Microsoft Excel (MS Office 2013). A positive signal was defined as a positive fluorescence intensity value after subtraction of the mean background intensity plus threefold standard deviation derived from the random peptides on each microarray slide. This yields a probability of >99 % of positivity for a positive signal [18]. Each of these positive signals was counted for the ADA positive sera as well as for the controls and plotted as frequency distribution against the IFX amino acid sequence to identify immunogenic IFX epitopes (Fig. 1). A sequence was defined as a relevant epitope when at least 50 % of the analysed sera were positive for the respective motif (i.e. at least 10 for IFX-treated patient sera and 7 for negative helathy control sera). In case of overlapping sequences, the “local most-recognized sequence” with the highest number of positive sera reactions was identified.

## Results

### Detection of ADA against infliximab in sera from IFX-treated patients

A heterogeneous bridging ELISA system was used to screen sera for ADA against infliximab. Sera were obtained from patients who experienced a suspected neutralization of drug efficacy during IFX treatment. 20 ADA-positive patients, 8 male and 12 female, aged from 4 to 80 years, were identified by the ELISA analyses. The specific ADA levels in those sera were in a concentration range of 14.4 µg/ml to more than 80 µg/ml with the latter being the upper limit of detection (Table 2). ADA concentrations of sera from 14 untreated healthy controls were consistently below the assay’s lower detection limit of 1.25 µg/ml.

### Distinct IgG epitopes of anti-infliximab-positive sera determined by oligopeptide microarray epitope mapping

**Fig. 2:**
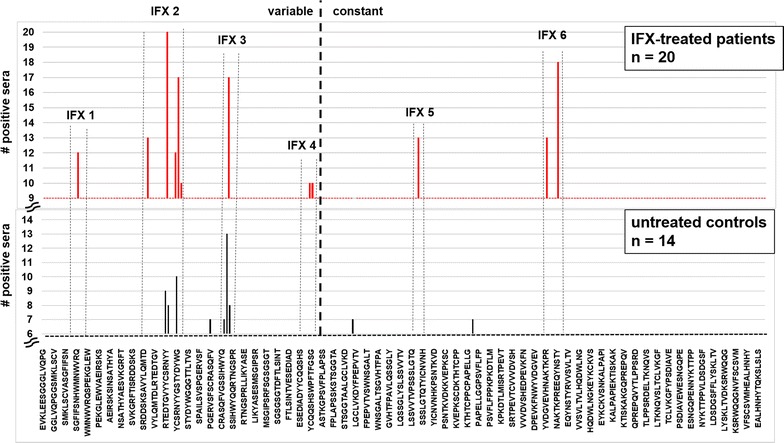
Epitope mapping of IFX peptide microarrays with ADA-positive patient sera. Six relevant epitopes were identified. An epitope was designated as relevant when at least 50 % of the analysed sera showed a positive reaction (Y axis scale adjusted accordingly). IFX epitope mapping was performed three times in duplicate. One representative result is shown

**Fig. 3:**
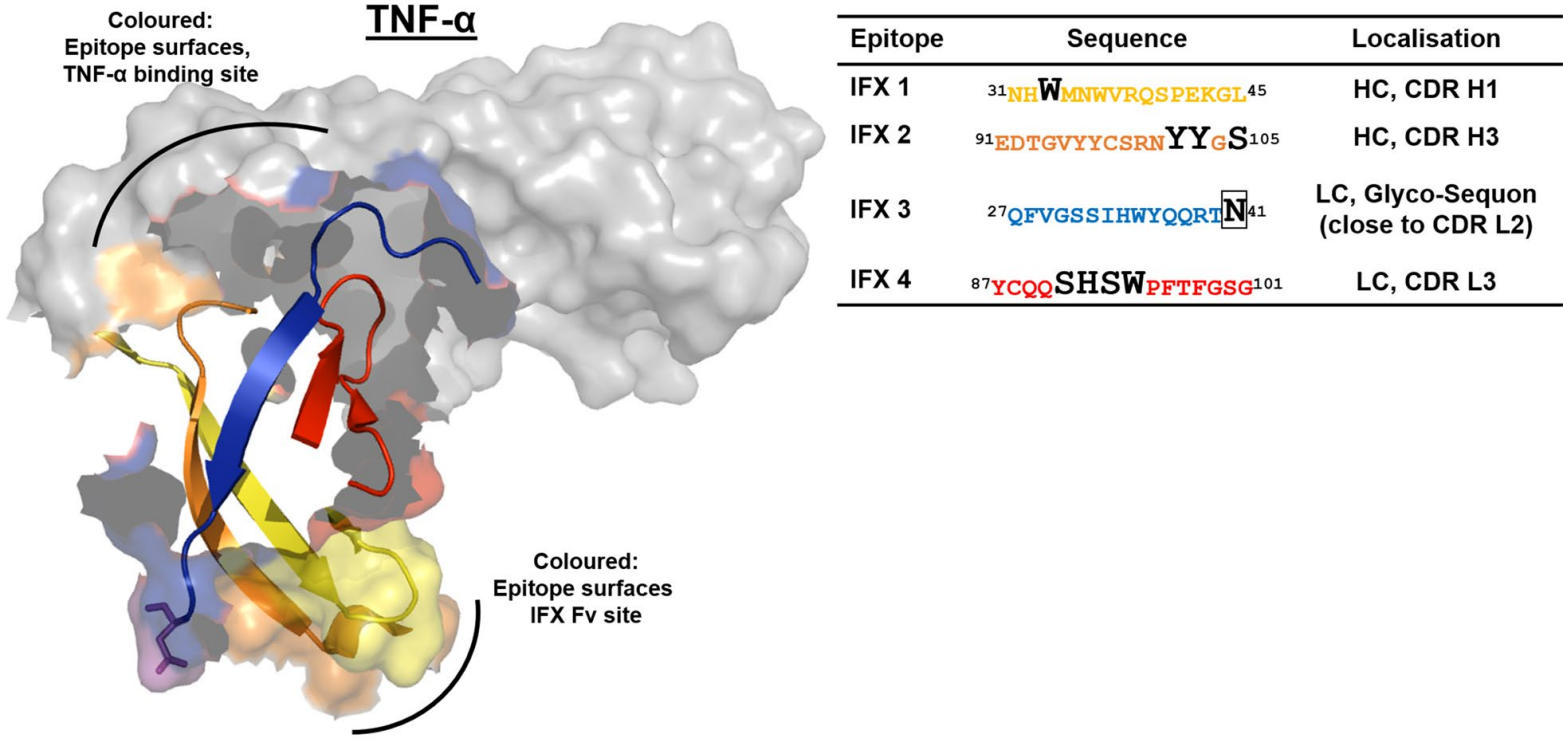
Structure of the IFX Fab fragment interaction with TNF-α. The identified IFX epitopes are located in or in close proximity to the IFX CDR involved in TNF-α binding. The mapped epitopes of the IFX variable segment of the heavy chain are indicated in* yellow* and* orange* (IFX 1 and 2), the light chain epitopes are shown in* blue* and* red* (IFX 3 and 4). The amino acids with direct contact to TNF-α in the CDR of IFX are indicated by* black letters* in the epitope sequences.* Boxed* N41 in epitope IFX 3 is part of a glycosylation sequon

To obtain information about immunogenic epitopes, an oligopeptide microarray epitope mapping system was established. The sequence of the variable region of IFX (drug bank acc. no. DB00065) along with the heavy chain constant region was synthesized chemically as 15-meric oligopeptides with an offset of two amino acids (13 amino acids overlap). These peptides were spotted onto a functionalized microarray slide. The microarrays were incubated with 20 ADA-positive and 14 control sera from untreated, healthy subjects each. Serum IgG binding was assessed with a fluorophore-labelled antibody against human IgG and analysed by fluorescence imaging. For positive signal identification a statistical procedure was used that warrants >99 % probability for a positive signal by comparison with the mean background intensity derived from random peptides on each slide [18]. Positive signals were compiled for all sera and plotted against the IFX sequence to identify relevant immunogenic IFX epitopes (Fig. 2). A peptide sequence was considered a relevant epitope if at least 50 % of the analysed sera were positive for this sequence. In case of overlapping sequences in a recognition cluster, the peptide sequence with the highest number of positive serum reactions was identified. Motif clusters that were designated as relevant epitopes are indicated by dashed lines (Fig. 2). Of the six relevant epitopes identified, four are located in the variable region of IFX (IFX 1–4) and two in the constant region (IFX 5 and 6). Epitopes IFX 1 and IFX 2 reside on the variable part of the heavy chain of IFX while IFX 4 is located in the variable light chain. These three epitopes are part of the complementary-determining region (CDR) of the IFX-TNF-α interaction site (Fig. 3) [19]. The light chain epitope IFX 3 is located close to CDR L3 and contains a glycosylation sequon at position N41. Interestingly, also epitope IFX 6 being situated in the constant region of the heavy chain contains a glycosylation sequon: the conserved human IgG1 glycosylation site N299. Epitope IFX 5 is also located in the constant region of the heavy chain. The binding frequencies of the patient sera against each epitope were varying: While IFX 4 just met the recognition frequency threshold of 50 % (10/20) with IFX-treated patient sera, IgG from all ADA sera bound to epitope IFX 2 (20/20, Fig. 3). Surprisingly, the recognition of IFX epitopes was not restricted to IFX-treated patient sera. Particularly pronounced recognition of epitopes by control sera occurred for IFX 2 and 3 with 9/14 and 13/14 control sera binding, respectively (Fig. 2).

### IgG from IFX-treated patient serum binds stronger to the identified IFX epitopes than IgG from untreated healthy controls

**Fig. 4:**
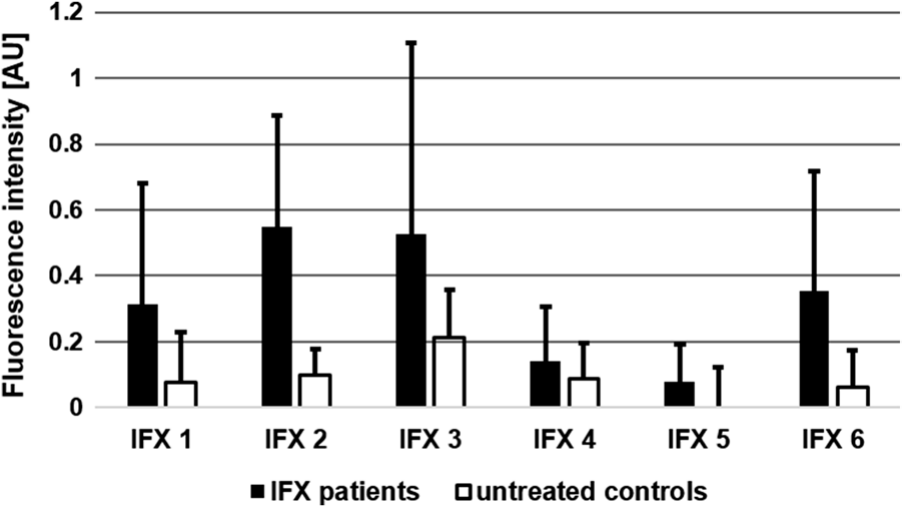
IgG antibodies from IFX-treated patient sera bind stronger to IFX epitopes than serum IgG from untreated healthy controls. Mean signal intensity of antibody-binding of IFX-treated patient serum and serum from untreated healthy controls to identified IFX epitopes (Fig. 2). Results of three independent epitope mapping experiments, each performed in duplicate, are shown. AU, arbitrary units

According to ELISA analyses, sera from IFX-treated patients resulted in a positive ADA ELISA signal, whereas untreated control sera were negative. Yet, some of the untreated control sera showed reactivity with certain IFX epitopes, especially pronounced against the epitopes IFX 2 and 3 which are located in the variable region of IFX. The question arose if there are differences in “patient epitopes” vs. “control epitopes”. In order to further specify the characteristics of the epitopes, a semi-quantitative analysis of the antibody binding strength in the microarray system was performed. For this, the mean fluorescence intensity obtained in the epitope mapping experiments for each relevant IFX epitope was calculated for the IFX-treated patient group and the untreated healthy controls (Fig. 4). The results show that antibody binding to the relevant epitopes is more pronounced in the IFX-treated patient sera than in the controls. This could be either due to a larger amount of ADA or a higher affinity of those antibodies. The stronger ADA array signals from IFX-treated patient sera as compared to the low signals from untreated controls is in line with the ADA ELISA results. According to the ELISA, high reactivity against the complete IFX molecule was observed in samples from IFX-treated patients as opposed to negative results with untreated healthy control sera.

### Anti-infliximab serum antibodies do not bind to the constant region of infliximab as determined by ELISA rheumatoid factor analysis

As the majority of IFX-treated patient sera recognize at least one epitope in the constant region (IFX 5 and/or 6) serum rheumatoid factors (RF) including isotypes IgM, IgA and IgG were analysed by a commercial RF ELISA system (Table 3). The test results were negative for all isotypes and sera except for patient sera 438 and 648 and control serum F 46 which showed moderate (30.7, 27.5 and 24.4 U/ml) IgG binding (Table 3). Serum F44 showed a positive IgA RF signal of 28.3 U/ml. However, this signal was not high above the threshold (25 U/ml) for IgA. IgM and IgG RF were negative for F44.

**Table 3:** Rheumatoid factor (RF) ELISA analysis of ADA-positive IFX-treated patients and ADA-negative untreated healthy control sera

Patient ID	RF (U/ml)			Control ID	RF (U/ml)		
	IgM	IgA	IgG		IgM	IgA	IgG
438	1.1	1.4	30.7	P*	2.0	3.4	8.4
452	5.1	2.8	5.0	F10	3.5	6.5	15.0
485	1.4	1.8	6.2	F22	1.9	2.3	14.2
522	1.7	3.2	16.4	F28	nd	nd	nd
587	3.3	2.0	3.6	F31	2.3	1.8	7.2
609	3.8	9.1	18.3	F32	3.1	18.9	5.7
623	3.1	8.1	10.2	F33	3.9	3.4	7.4
626	2.9	8.2	9.4	F34	1.3	2.4	8.4
638	2.7	2.8	4.7	F38	2.8	5.2	10.3
639	2.7	2.3	5.0	F41	2.2	2.0	10.3
648	2.3	11.4	27.5	F42	1.5	3.2	13.1
652	1.9	7.8	12.3	F44	3.5	28.3	11.3
660	2.2	3.4	12.3	F45	0.9	0.2	3.4
681	1.3	12.2	8.7	F46	2.2	3.5	24.4
694	2.9	3.3	10.2	F47	nd	nd	nd
695	2.9	4.0	14.5				
719	1.1	8.8	7.4				
721	2.7	0.9	4.1				
722	4.4	4.5	3.3				
724	0.9	21	4.8				

RF ELISA results are considered being negative when below 10 U/ml for IgM, 25 U/ml for IgA, and 20 U/ml for IgG antibodies

*nd* not determined

### No cross-reaction of anti-infliximab serum antibodies with adalimumab as determined by anti-adalimumab ELISA

As development of ADA may entail termination of an IFX therapy, it is important to know whether a switch to other TNF-α-blockers such as adalimumab (ADL) is feasible. To address this question, the reactivity of the IFX-treated patient sera to ADL was analyzed in an anti-ADL antibody ELISA assay (Table 4). As a result, none of the IFX-treated patient sera showed any anti-ADL antibody content above the assay’s detection limit (1.25 µg/ml). Control sera were also consistently negative for anti-ADL antibodies (<1.25 µg/ml).

**Table 4:** Cross-reactivity analysis of ADA

Patient ID	Age	Anti-ADL (µg/ml)	Control ID	Age	Anti-ADL (µg/ml)
438	29	<1.25	P*	48	<1.25
452	30	<1.25	F10	20	<1.25
485	64	<1.25	F22	25	<1.25
522	42	≤1.25	F28	22	<1.25
587	41	<1.25	F31	45	<1.25
609	30	<1.25	F32	59	<1.25
623	57	<1.25	F33	25	<1.25
626	57	<1.25	F38	53	<1.25
638	32	<1.25	F41	19	<1.25
639	33	<1.25	F42	32	<1.25
648	4	<1.25	F43	31	<1.25
652	31	<1.25	F44	44	<1.25
660	26	<1.25	F45	36	<1.25
681	?	<1.25	F46	35	<1.25
694	13	<1.25	F47	33	<1.25
695	37	<1.25			
719	16	<1.25			
721	?	<1.25			
722	42	<1.25			
724	80	<1.25			

IFX-treated patient sera positive for anti-IFX antibodies and untreated healthy control sera were analyzed in an ELISA system for anti-adalimumab (ADL) antibody detection

## Discussion

Several studies have shown that anti-drug-antibodies (ADA) are induced in a substantial number of patients who are treated with therapeutic monoclonal antibodies. It is assumed that the formation of ADA in such patients is responsible for several side effects reported for therapeutic antibodies [20, 21]. Whether or not ADA develop in a patient is dependent on various factors such as the therapeutic antibody itself, application route, dosage, frequency of application, and the immunological history of patients with or without co-medication [1, 2, 4, 5, 15, 22]. All of these factors are classical parameters in vaccine development—not surprisingly, since ADA induction can be considered as an undesired immunization event. A key parameter in every immunization/vaccination effort is the immunogenicity of the antigen administered. Immunogenicity is governed by the physical status of the antigen (aggregate/monomer), its valency (mono- or multivalent) and the presence of B and T cell epitopes. In addition, cross-recognition caused by pre-existing immunity against a similar antigen may also play a role.

This study aims to shed light on the immunogenicity of the therapeutic anti-TNF-α-antibody IFX, a widely used drug for the treatment of TNF-α-dependent autoimmune disorders. We wanted to identify the most prominent immunogenic elements of IFX by using ADA-containing patient sera along with a chip-based oligopeptide epitope mapping technology. In contrast to a previous study where linear peptide motifs were allowed to adsorb directly onto a solid support—a situation notorious for low sensitivity—and which was performed with processed patient sera [23], our oligopeptide microarray epitope mapping system used cellulose-anchor equipped peptides instead and operated with non-processed patient sera. This more physiological setup revealed six prominent linear epitopes on IFX (Fig. 2). Two of these epitopes are located in the Fc part of the immunoglobulin in the constant region of the heavy chain (IFX 5 and 6) which is of human IgG1-origin. As humanization is considered the silver bullet to low immunogenicity of antibody drugs, this observation is disconcerting at first glance. However, the existence of immunoglobulin allotypes and the occurrence of auto-antibodies against the constant regions of immunoglobulins (rheumatoid factors; RF) substantiate the conceivability for such a type of reaction. In fact, serum antibodies against the constant region of the heavy chain of IFX have been described [24, 25]. On the other hand, both epitopes IFX 5 and 6 might not be accessible for antibody-binding on intact, correctly folded IFX [26], and—furthermore—IFX 6 contains the conserved IgG1 HC glycosylation site and thus may be camouflaged by the attached glycan on IFX in its native state. The almost complete absence of RF in the tested IFX-treated patient sera supports these hypotheses. The four other identified epitopes are located in the variable region of IFX. This result is not surprising as potentially immunogenic murine peptide sequences are located in this area. According to our study, ADA-binding occurs most frequently in the CDRs of IFX. Although this is highly unfortunate from the clinical point of view, it provides a conclusive explanation for the observed decline in IFX efficacy upon ADA formation. Epitopes IFX 1, 2 and 4 display amino acids which are directly involved in TNF-α-binding. Binding of ADA to these peptides will most certainly impair the interaction of IFX with TNF-α. Consequently, the therapeutic efficacy of IFX may decline along with an increase in ADA [11]. Of special interest as a clinical biomarker for adverse patient reactions may be epitope IFX 2 as all of the investigated patient sera contained antibodies against this epitope. Epitope IFX 3, which is also located in the variable region, is also interesting in several respects: Even though it is not directly involved in the TNF-α–IFX-interaction, its vicinity to CDR L2 may be sufficient to interfere with TNF-α-binding. While in the case of IFX 1, 2 and 4 the ADA may compete directly with TNF-α in IFX-binding, the mode of action of ADA bound to IFX 3 may be one of steric hindrance. The presence of an N-glycosylation sequon in IFX 3 is another interesting feature. We can only speculate whether or not this site is glycosylated in the native IFX antibody. Indications are that this site may not be glycosylated [26]. As the non-glycosylated peptide IFX 3 is recognized by 17 out of 20 patient sera, glycosylation at this position is not a prerequisite for recognition by ADA. Yet, it could still be possible that an N-glycan structure, which is part of IFX 3 in the native molecule, is co-recognized by ADA, and that ADA-reactivity towards this epitope would be markedly increased upon glycosylation. The non-glycosylated epitope IFX 3 is also recognized by a high number of control sera. Although the healthy individuals providing those sera have not been treated with IFX, they obviously harbour antibodies against this motif. One possible explanation for this phenomenon would be an immunogenetic/immunologic predisposition, i.e. a high rate of anti-IFX 3 paratopes in the naive B-cell repertoire or a pre-existing immunity against the peptide sequence (or a very similar one) of IFX 3. The question of a potential predisposition against therapeutic antibodies is still a subject of debate [8, 13, 14]. In this context, one interpretation of our results would be an IFX-independent, pre-existing immunity against a peptide sequence related to IFX 3. Likewise, a potential pre-existing immunity against IFX 2 and a subsequent boost of this reactivity upon IFX treatment is in line with our data. Such an explanation would also be supported by the different affinities of the “patient” and “control” antibodies against these two epitopes, as indicated by the differing fluorescence signal intensities (Fig. 4). The latter may also explain the negative ADA ELISA results for the untreated healthy control sera in contrast to the positive results obtained with the IFX-treated patient sera. However, regarding pre-existing antibodies, the limit of detection of the used anti-IFX antibody ELISA detection system has to be taken into account. The sensitivity of the system is rather low (<1.25 µg/ml) compared to other ELISA systems (25–500 ng/ml), but thus also leads to a lower rate of false positive results. That means that only patients with a considerable amount of anti-IFX antibodies are diagnosed as ADA positive. A disadvantage is the difficult detection of pre-existing low level ADA or the monitoring of the ADA development from low amounts to higher serum levels. It has to be mentioned that ADA of the subclass IgG4 cannot be detected by the bridging ELISA ADA detection system used in this study. Due to an intermolecular Fab arm exchange IgG4 is monovalent. Yet, this ELISA system reflects the present diagnostic situation for the detection of anti-drug antibodies (ADA).

## Conclusions

Our data indicate a host-borne drug neutralization by ADA which are directed against the CDR of IFX, i.e. the TNF-α-binding regions. Those sites are not present in other anti-TNF-α antibodies. The results of this study shall lead to a better understanding of the development of anti-biological antibodies and to a knowledge-based suggestion for a safe and efficacious switch to another antibody during anti-TNF-α therapy. In this context, the oligopeptide microarray technique could become a useful tool for treatment monitoring with a special emphasis on the early detection of ADA development to biologicals. The screening platform may lead to the development of less immunogenic therapeutic antibodies in the future from which patients will benefit in terms of reduced adverse events and longer lasting drug effects.

## Abbreviations

ADA: anti-drug antibodies
ADL: adalimumab
CDR: complementary-determining region
ELISA: enzyme-linked immunosorbent assay
HC: heavy chain
IBD: inflammatory bowel disease
IFX: infliximab
LC: light chain
RA: rheumatoid arthritis
RF: rheumatoid factor
TNF: tumor necrosis factor
